# Robotic stereotactic body radiotherapy for the management of adrenal gland metastases: a bi-institutional analysis

**DOI:** 10.1007/s00432-022-03943-0

**Published:** 2022-03-15

**Authors:** Felix Ehret, David Kaul, Markus Kufeld, Clara vom Endt, Volker Budach, Carolin Senger, Christoph Fürweger, Alfred Haidenberger, Alexander Muacevic

**Affiliations:** 1 Berlin Institute of Health at Charité—Universitätsmedizin Berlin, 10117 Berlin, Germany; 2 Department of Radiation Oncology, Corporate Member of Freie Universität Berlin and Humboldt-Universität zu Berlin, Charité—Universitätsmedizin Berlin, 13353 Berlin, Germany; 3 European Radiosurgery Center, 81377 Munich, Germany; 4 Charité CyberKnife Center, Corporate Member of Freie Universität Berlin and Humboldt-Universität zu Berlin, Charité—Universitätsmedizin Berlin, 13353 Berlin, Germany; 5 German Cancer Consortium (DKTK), German Cancer Research Center (DKFZ), Partner Site Berlin, 69120 Heidelberg, Germany; 6 Department of Stereotaxy and Functional Neurosurgery, University Hospital Cologne, 50937 Cologne, Germany

**Keywords:** Adrenal gland metastasis, Adrenal gland, Metastasis, Stereotactic body radiotherapy, SBRT, Oligometastases

## Abstract

**Purpose:**

Adrenal gland metastases (AGMs) are a common manifestation of metastatic tumor spread, especially in non-small cell lung cancer (NSCLC) and small cell lung cancer (SCLC). In patients with a limited systemic tumor burden, effective treatments for AGMs are needed. Due to varying fractionation schemes and limited reports, short-course treatment results for stereotactic body radiotherapy (SBRT) for AGMs are lacking. This work analyzes the outcomes of short-course SBRT for AGMs.

**Methods:**

Patients who underwent robotic SBRT for AGMs with one to five fractions were eligible for analysis.

**Results:**

In total, data from 55 patients with 72 AGMs from two institutions were analyzed. Most AGMs originated from renal cell carcinoma (38%) and NSCLC (35%). The median follow-up was 16.4 months. The median prescription dose and isodose line were 24 Gy and 70%, respectively. Most patients (85%) received SBRT with just one fraction. The median biologically effective dose assuming an α/β ratio of 10 (BED_10_) was 80.4 Gy. The local control and progression-free survival after 1 and 2 years were 92.9%, 67.8%, and 46.2%, as well as 24.3%, respectively. Thirteen patients (24%) suffered from grade 1 or 2 toxicities. The BED_10_ showed a significant impact on LC (*p* < 0.01). Treatments with a BED_10_ equal to or above the median were associated with a better LC (*p* < 0.01).

**Conclusion:**

Robotic SBRT is an efficient and safe treatment modality for AGM. Treatment-associated side effects are sporadic and manageable. Results suggest short-course SBRT to be a preferable and time-saving treatment option for the management of AGMs if an adequate BED_10_ can be safely applied.

## Introduction

Given their rich sinusoidal blood supply, the adrenal glands are a frequent target of metastatic tumor spread (Kung 1990; Lam 2002). Metastases to this location usually originate from lung cancer, including both non-small cell lung cancer (NSCLC) and small cell lung cancer (SCLC), but may also be caused by gastric, esophageal, and pancreatic cancer, as well as renal cell carcinoma (Lam 2002). Adrenal gland metastases (AGMs) can be symptomatic depending on their size and extent and may even cause life-threatening complications in case of an adrenal crisis (Kung 1990). However, only approximately 4% of AGMs are symptomatic (Lam 2002). With recent advances in imaging and standardized tumor staging in cancer patients, AGMs are more likely to be detected at an early stage of the disease long before they cause any symptoms (Allen 2015; Kim 2018; Mayo-Smith 2001).

Recently, our understanding of metastatic cancer, and to some extent even its definition, has evolved and changed (Guckenberger 2020). Patients suffering from a limited amount of metastatic spread—usually up to five metastases in up to three organs—can be considered to be in an “oligometastatic” state of disease (Hellman 1995; Lievens 2020; Milano 2021; Weichselbaum 2011). Such patients may profit from an ablative therapy of their metastatic lesions, ideally leading to a prolonged progression-free survival (PFS) and overall survival (OS) as recently shown (Gomez 2016, 2019; Palma 2019). Previous studies investigated the efficacy and safety of stereotactic body radiotherapy (SBRT) for the treatment of AGMs and found favorable results, making SBRT a proper treatment modality for delivering ablative treatments (Buergy 2021; Chen 2020; Haidenberger 2017; König 2020; Plichta 2017; Scouarnec 2019; Zhao 2020). However, fractionation schemes of reported cohorts are quite heterogeneous, with many patients receiving five or more fractions (Buergy 2021; Chen 2020). Besides, the ideal biologically effective dose (BED) to achieve local control (LC) remains unclear. Still, recent findings and analyses suggest that a BED_10_ of 80 Gray (Gy) or more may be preferable (Buergy 2021; Chen 2020; Zhao 2020). A recent review recommended a BED_10_ of approximately 116 Gy to achieve 1-year LC rates of 95% or more (Stumpf 2021). The objective of this bi-institutional analysis is to report the efficacy and safety of robotic SBRT executed with a maximum of five fractions and to investigate how the applied BED influences the LC.

## Materials and methods

Patients between 2005 and 2021 who were treated with robotic SBRT for an AGM at two institutions were eligible for analysis. A histopathologically confirmed diagnosis of a malignancy before SBRT was required. AGM diagnosis was either made utilizing computed tomography (CT), magnetic resonance imaging (MRI), biopsy, or positron emission tomography (PET) combined with CT (PET–CT). Patient and treatment data were retrospectively analyzed to confirm study eligibility. All patients underwent SBRT utilizing a CyberKnife^®^ (CK) robotic radiosurgery system (Accuray Inc., Sunnyvale, CA, USA). All but one patient received a percutaneous placement of one gold fiducial into or close to the AGM. Subsequent planning imaging with contrast-enhanced CT was acquired. Treatment planning, dose constraints, movement compensation, and treatment delivery were executed and taken into account as previously described (Haidenberger 2017). For motion compensation, the Synchrony^®^ respiratory tracking system (Accuray Inc., Sunnyvale, CA, USA) continuously synchronizes the delivery of the treatment beam to the AGM moving with respiration by generating a correlation model between the breathing pattern of the patient monitored in real time and the position of the gold fiducial at different points in the respiratory cycle. One applied treatment margin was not assessable due to a software error. Only treatments with up to five fractions were included. LC was defined as an unchanged or decreased AGM volume on follow-up imaging with CT or MRI. Local failure (LF) was defined as the absence of LC on follow-up imaging assessed by a board-certified radiologist. Every treatment was independently integrated in the LC analysis. Only AGM treatments with at least one radiographic follow-up were included into the LC analysis. LC, PFS, and OS were assessed utilizing the Kaplan–Meier estimator, starting from the first day of SBRT. In accordance with previous studies, an AGM α/β ratio of 10 was assumed (Buergy 2021; König 2020; Scouarnec 2019). Subsequently, we reported the respective BED_10_ following the standard linear-quadratic formula. Grading of treatment-associated toxicities followed the Common Terminology Criteria for Adverse Events (CTCAE), version 5.0. Descriptive statistics utilized ranges, median, and mean for continuous variables. For categorical variables, the respective frequencies with percentages were reported. Differences in time-to-event variables were evaluated using the log-rank test. Tests for the proportional-hazards assumption were based on Schoenfeld residuals. All *p*-values were two-sided. The statistical significance was defined as a *p*-value ≤ 0.05. Statistical analyses were performed with STATA MP 16.0 (StataCorp, College Station, TX, USA).

## Results

### Patient characteristics

A total of 55 patients with 72 AGM treatments were identified. Forty AGMs (56%) were right-sided; the remaining 32 metastases were located on the left (44%). A total of 14 patients (25%) were treated for more than one AGM during the course of their disease. The median age at treatment was 66.3 years and the median Karnofsky performance status (KPS) was 90%. Most AGMs originated from lung tumors, including NSCLC (19 patients, 35%) and SCLC (four patients, 7%). The second most frequent primary tumor was the renal cell carcinoma (21 patients, 38%). Thirty-eight cases (53%) had received a systemic treatment in the period of up to three months until the respective SBRT, most of them receiving chemotherapy (18 cases, 47%). At the time of SBRT, 60% of patients were suffering from metastatic spread to at least one more organ other than the adrenal gland. SBRT was the primary treatment modality for 59 AGMs (82%). The remaining 13 metastases (18%) were recurrences. Patient characteristics are summarized in Table 1.

**Table 1:** Patient and treatment characteristics

Total number of patients	55
Total number of AGMs	72
Sex, male (%)/female (%)	39 (71)/16 (29)
Tumor location, left (%)/right (%)	32 (44)/40 (56)

	Median	Mean	Range
Age (years)	66.3	65.7	43.8–85.3
Pretreatment KPS (%)	90	91.3	70–100
Follow-up (months)	16.4	24.1	0.2–107.1
Gross tumor volume (cc)	16.8	21.1	2.1–71.6
Planning target volume (cc)	38.3	40.8	4.9–95.9
Prescription dose (Gy)	24	24.5	19–45
Fractions	1	1.3	1–5
Prescription isodose line (%)	70	68.7	60–70
BED_10_ (Gy)	80.4	75.1	43.2–112.5
Conformity index	1.1	1.1	1.02–1.46
Homogeneity index	1.4	1.4	1.43–1.67
Coverage (%)	97.9	96.6	85.0–100

Tumor entities	Number of patients
Renal cell carcinoma (%)	21 (38)
NSCLC (%)	19 (35)
SCLC (%)	4 (7)
Other (%)	11 (20)

*KPS* Karnofsky performance status, *cc* cubic centimeters, *Gy* gray, *BED*_*10*_ biologically equivalent dose (α/β ratio = 10), NSCLC non-small cell lung cancer, *SCLC* small cell lung cancer

### Treatment characteristics

The median gross tumor volume (GTV) and planning target volume (PTV) were 16.8 and 38.3 cubic centimeters (cc) respectively; the latter was created by adding a median (mean) safety margin of 5 (3.8) mm to the GTV. The median prescription dose and isodose line were 24 Gy and 70%, respectively. Single-fraction prescription doses ranged from 19 to 25 Gy. The median and mean prescribed BED_10_ were 80.4 and 75.1 Gy, respectively. Sixty-one treatments (85%) had a BED_10_ ranging between 70 and 90 Gy. Sixty-one AGMs (85%) had been treated with one fraction, ten metastases (14%) had received three fractions, and one patient had received five fractions (1%). Prescription doses for fractionated treatments ranged from 24 to 45 Gy. The median conformity and homogeneity indices were 1.1 and 1.4, respectively. A median coverage of 97.9% was achieved in this series. All but one AGM treatment had utilized fiducial tracking with the Synchrony^®^ respiratory tracking system (Accuray Inc., Sunnyvale, CA, USA), one small metastasis had been planned with an internal target volume (ITV). Treatment characteristics are summarized in Table 1.

### Treatment outcomes, survival, and toxicity

**Fig. 1:**
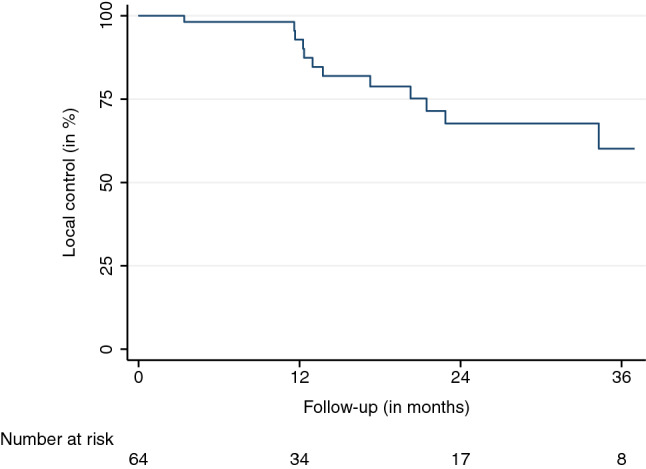
Local control

**Fig. 2:**
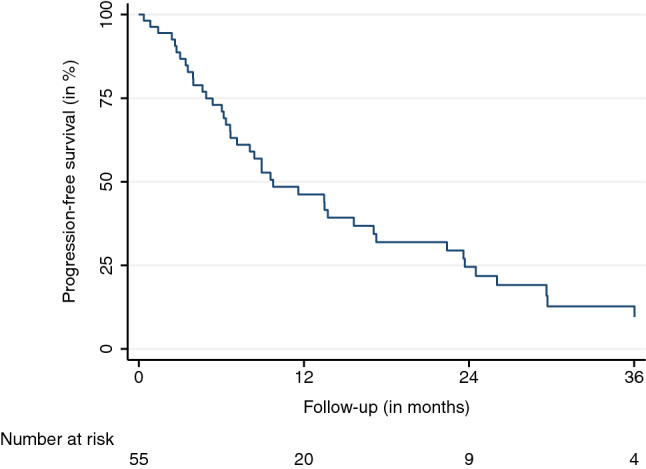
Progression-free survival

**Fig. 3:**
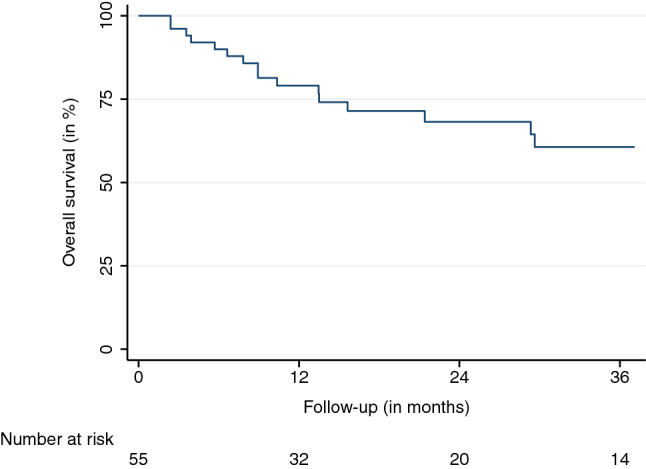
Overall survival

**Fig. 4:**
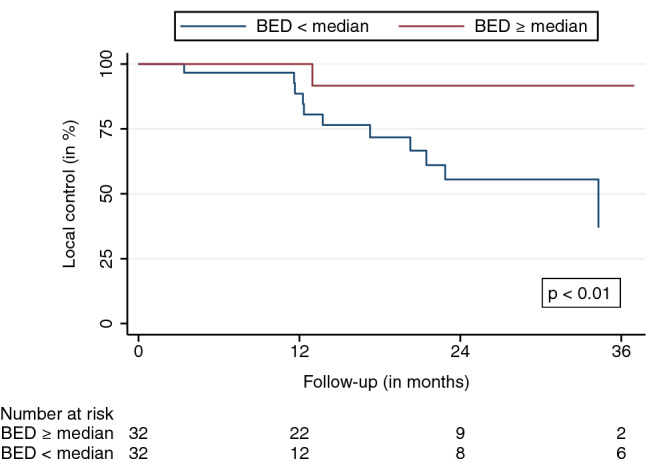
Local control stratified by the median BED_10_

**Fig. 5:**
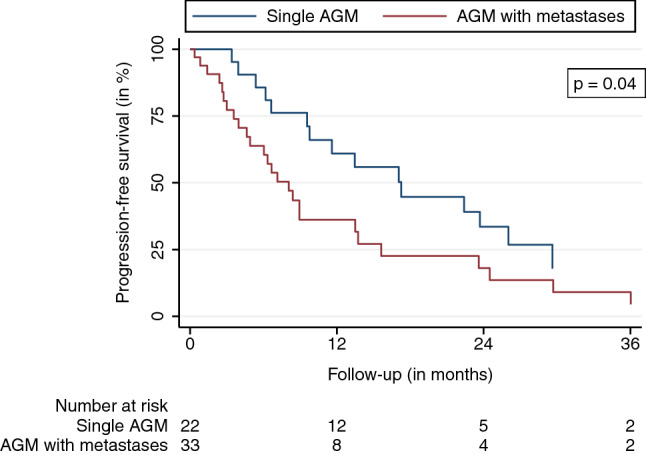
Progression-free survival stratified for patients with a single AGM at the time of SBRT versus those with AGM and additional metastases

The mean and median follow-up durations, beginning on the first day of SBRT, were 16.4 and 24.1 months, respectively. Eight (11%) AGMs did not have a radiographic follow-up before the patients’ transition to best supportive care, death, or being lost to follow-up. The LC at the last available follow-up was 79.6%. LC rates after 6, 12, 18, and 24 months were 98.1%, 92.9%, 78.8%, and 67.8%, respectively (95% confidence interval (CI) LC 12 months: 82.7–98.0, 95% CI LC 24 months: 49.8–81.4) (Fig. 1). Thirteen LF were observed. The median and mean BED_10_ for LF were 70 and 66 Gy. The median time to LF was 13.7 months. The observed PFS after 6, 12, 18, and 24 months were 73.0%, 46.2%, 31.9%, and 24.3%, respectively (95% CI PFS 12 months: 32.9-59.9, 95% CI PFS 24 months: 13.2-38.1) (Fig. 2). The median time to progression was 8.2 months. Distant progress was the primary reason for progression in the majority of patients (88%). In regard to the OS, 35 patients (64%) were alive at the last available follow-up and 20 had died (36%). OS rates after 6, 12, 18, and 24 months were 90.0%, 79.1%, 71.4%, and 68.3%, respectively (Fig. 3) (95% CI OS 12 months: 65.0-88.3, 95% CI OS 24 months: 51.6-79.9). Various patient, tumor, and treatment characteristics were analyzed for their impact on LC, PFS, and OS. The BED_10_ showed a significant impact on LC in the multivariable analysis after adjustment for GTV, applied PTV margin, and coverage (hazard ratio 0.85, *p* < 0.01). AGMs receiving more than the median BED_10_ showed a significantly improved LC (*p* < 0.01) (Fig. 4). Other factors did not impact LC. A significant PFS difference between single AGM and patients with additional metastases besides the treated AGM was detected (*p* = 0.04) (Fig. 5). However, this difference did not translate into an improved OS for patients with just one AGM (*p* = 0.74). No significant variables for the OS were identified.

In regard to the treatment toxicity, a total of 13 patients (24%) had side effects potentially associated with AGM SBRT. The most commonly observed toxicities were low-grade (1 and 2) nausea (five patients, 9%) and low-grade (1 and 2) fatigue (four patients, 7%). No treatment-related toxicities ≥ grade 3 were observed. In case of three patients (5%), who had only one remaining adrenal gland after surgical resection for metastasis, SBRT of the AGM of the contralateral gland caused adrenal insufficiency grade 2. In these patients, close monitoring of the hormone status before and after treatment delivery was indicated. One patient (2%) developed another adrenal insufficiency after SBRT in the presence of metastatic destruction of the contralateral adrenal gland. Due to a significantly decreased hormone production, all four patients had to start with hormone replacement therapy. No adrenal crisis occurred throughout the available follow-up of the affected patients.

## Discussion

Herein, we report one of the most extensive series of patients treated with robotic SBRT for AGMs. In particular, this bi-institutional analysis includes the largest series of patients treated with just one fraction. With more and more published studies, analyses, reviews, and a comprehensive meta-analysis of SBRT for AGMs, the discussion on ideal fractionation schemes and the respective BED continues (Buergy 2021; Stumpf 2021; Zhao 2020). In general, SBRT can be considered as an efficient and safe treatment modality for AGMs (Chen 2020). However, due to the limited size of the published cohorts and their heterogeneity, our knowledge in terms of patient selection and optimal dosimetric parameters remains limited (Buergy 2021; Chen 2020; Stumpf 2021). With a paradigm shift in oncology toward potentially curative treatment approaches in patients with limited metastatic spread, more work, especially of prospective nature, is necessary (Gomez 2019; Lievens 2020; Palma 2019; Weichselbaum 2011). In contrast to the available literature, our series primarily consists of patients treated with just one fraction, applying a median BED_10_ of 80.4 Gy (Buergy 2021; Chen 2020; Ippolito 2015). Only nine patients had received three fractions, and just one patient had been treated with five fractions out of a cohort of 55 patients. With LC rates of 92.9% and 67.8%, after 1 and 2 years, our reported results match remarkably well with the findings of a recent comprehensive meta-analysis of SBRT for AGMs (Chen 2020). In this meta-analysis of 39 studies, a BED_10_ of 60, 80, and 100 Gy corresponded to a 1-year LC rate of 70.5%, 84.8%, and 92.9%, and to a 2-year LC rate of 47.8%, 70.1%, and 85.6%, respectively (Chen 2020). Moreover, a recent study modeling the AGM control probability after SBRT found a BED_10_ of 116.4 Gy to achieve a 1-year LC rate of 95% (Stumpf 2021). With respect to these findings, it seems that dose escalation may play a crucial role for AGM SBRT, potentially explained by the abundance of lung and renal cell cancer histologies and the respective tumor biology (Chen 2020). Herein, we did not find any other significant predictors of the LC besides BED_10_. Neither tumor size, including GTV and PTV, histology, or other dosimetric parameters were found to play a decisive role. Despite an extensive sample size, our cohort still may not be large enough to identify underlying relationships on this matter. A distinct patient heterogeneity may also partially account for this. However, these findings are also in agreement with the reports of other studies (Chen 2020; König 2020; Zhao 2020). Scouarnec et al. reported three LF and discussed whether the high PTV and subsequently reduced coverage to protect organs at risk OAR may have played a role (Scouarnec 2019). With this being said, the assumed dose escalation to achieve a reasonable LC can only be achieved if OAR can be adequately saved from high doses. In general, SBRT for AGM can be delivered with a manageable toxicity (Chen 2020). According to the meta-analysis by Chen and colleagues, toxicities ≥ grade 3 are rarely reported (1.8%) (Chen 2020). Grade 1 and 2 toxicities mostly consist of nausea, vomiting, diarrhea, pain, and fatigue (Haidenberger 2017; König 2020; Scouarnec 2019; Zhao 2020). These previously reported findings are in accordance with our results. Notably, we observed an adrenal insufficiency grade 2 in four patients after robotic SBRT. Three out of these patients had contralateral adrenalectomy, greatly increasing the risk for hormone deficiencies after treatment. We suggest monitoring adrenal hormone production in surgically pretreated or preirradiated patients to prevent adrenal crises, which have not been explicitly reported in the context of SBRT yet (Chen 2020). Overall, our series provides the first extended SBRT cohort for AGMs, mostly utilizing single-fraction treatments. The results are comparable to the available literature. We suggest using such single-session treatments if the AGM volume and respective anatomy with neighboring OAR allow for the application of a decent BED_10_. Due to the retrospective nature of this work, respective biases and limitations may be apparent. For example, targeted therapies and immunotherapies can play a role in the LC of AGMs and PFS as well as OS—especially in patients with lung cancer and renal cell carcinoma. However, given our study cohort and potential sampling biases, underlying treatment effects may remain undetected. Moreover, differences in the radiosensitivity of the included tumor entities and respective potential for dose de-escalation could go unnoticed as well. Despite the relatively large number of patients treated with five or fewer fractions, the overall sample size is still limited to draw further conclusions. Finally, with technical changes and updates during the past years of treating patients with robotic SBRT, an impact on the treatment quality over time cannot be explicitly excluded.

## Conclusions

Robotic SBRT is an efficient and safe treatment modality for AGMs. Treatment-associated side effects are sporadic and manageable. Results suggest short-course SBRT to be a preferable and time-saving treatment option for the management of AGMs if a BED_10_ of more than 80 Gy can be safely applied.

## Data Availability

The data that support the findings of this study are available from the corresponding author, F.E., upon reasonable request.
